# Definitions

**Published:** 1883-02

**Authors:** E. W. Berridge


### DEFINITIONS.

E. W. Berridge, M. D., M. I. H. A.

It is an extremely difficult task to define both accurately and concisely and to so express a truth that the spirit and letter of the statement are perfectly harmonious. On recently placing the resolutions of the I. H. A. before one of our oldest Hahnemannians, he pointed out that there was an ambiguity with regard to two of them, and that though he fully agreed with their spirit and real intention, yet he could not subscribe to them until they had been more clearly and definitely expressed. As it is desirable that there should be no misunderstanding of the principles which we maintain, I shall propose the following verbal alterations at our next meeting, which I trust will be accepted.

The first clause of our preamble and resolutions, adopted at Milwaukee in 1880, reads thus:

"We believe the *Organon* of the healing art, as promulgated by Samuel Hahnemann, to be the only reliable guide in Therapeutics."

But the question has been asked: "What is meant by Therapeutics?" As this is a medical term, its use should be determined by the voice of the profession. As it is originally a Greek word, the profession should use it only in the sense in which the great Greek physicians used it. On referring to Liddell and Scott's Greek lexicon I find that Hippocrates used it in the sense of "healing *by medicine*." *Later* it was used in the sense of healing generally by any means. The former is, therefore, the true signification of the word; and Carroll Dunham was quite right when he defined Homœopathy as the science of *Therapeutics*, considered as distinct from *Hygiene*, which belongs to no special system of medicine. Yet, as the word has been popularly used in a double sense, we ought to clearly define it, because, though if we interpret it strictly, our declaration is true —if we interpret it widely, it is not true, seeing that HAHNEMANN did not give us in his *Organon* a complete system of Hygiene, but only of the art of healing by medicine, the former being but briefly touched upon.

I propose, therefore, that this first clause should be thus amended:

"We believe the *Organon* of the healing art, as promulgated by Samuel Hahnemann, to be the only reliable guide in Therapeutics —that is, the art of healing by medicines."

I will here point out that I suggest the term *art* in preference to *science*, because the *art* contains the practical rules, the *science* the philosophical explanation of those rules, and this has never been yet given in its completeness, HAHNEMANN considering it a matter of little consequence.

The seventh clause reads: "That in non-surgical cases we disapprove of medicated topical applications and mechanical appliances as being also non-homœopathic."

This resolution, though perfectly true in the spirit and intention in which it was framed, is yet, as it stands, capable of an erroneous and untrue interpretation.

In the first place, let us define "surgical" cases. "Surgery" (*chirurgia*) is the art of healing by *manual* interference, just as "medicine" is the art of healing by physic. Therefore surgical cases are those which require only manual aid; medical cases are those which require medical aid. A fractured bone is a surgical case; a paralytic attack belongs to medicine. On the other hand, there is a class of cases which belong to neither division exclusively. Thus, the fracture of a bone may excite so much fever that medicine must be given, and a paralyzed limb may be benefited by friction or other manipulations. Now, we see the extreme importance of an accurate definition. Our resolution declares that in all non-surgical cases medicated topical applications and mechanical appliances are equally forbidden, thereby implying that in surgical cases they are equally permissible. Under which class shall we place varicose veins of the lower limb? They depend upon constitutional causes, and hence must be treated medically; yet they may be temporarily relieved by mechanical appliances, such as an elastic stocking. If we say that they are surgical cases we thereby permit the use of external medicated applications, which is contrary both to HAHNEMANN's teaching and to a sound pathology; if we call them medical, then the letter (though not the spirit and intention) of this resolution precludes us from relieving them by a bandage.

Before taking such an important step as altering our already accepted resolutions, it may be well to see what interpretation has already been placed upon them by those who originally formulated them. Dr. Pearson, in his excellent presidential address in 1882, illustrated this resolution by quoting HAHNEMANN's denunciation of the "criminal practice" of "destroying the granulations of sycosis by ligature, excision, or the application of a hot iron." But this does not solve the verbal difficulty, because the master's protest is here directed against surgical operations which removed the objective symptom, and our resolution, as at present worded, refers to mechanical appliances only. There is a considerable difference between the two; to ligate enlarged veins is a surgical operation; to apply a bandage to them is to employ a mechanical appliance; both are imperfect, so far as they do not touch the original seat of the disease; but the latter is merely negative, whereas the former is dangerous, and so at times positively injurious.

When this resolution was framed, I remember that Professor H. C. Allen proposed the introduction of the clause about mechanical appliances as a protest against the practice of those who resorted to pessaries *instead of* curing the prolapsus by the *simillimum*. I think here we have the true sense of the resolution, which needs only a verbal modification to fully express that sense without danger of cavil or misconstruction. What every Hahnemannian desires to express is (1) that in no case of disease (but only in cases of injury) is it good practice to use medicated topical applications; (2) that in no case of disease (but only in cases of injury) is it good practice to resort to surgical operations or mechanical appliances, *in the place of the best indicated remedy*, thus leaving it for the intelligent and scientific physician to decide when and how far manual interference may aid the remedy without doing any mischief at the same time. To take the case of cataract: the true homœopathician will never at once resort to an operation. He will first administer that remedy which from time to time is most homœopathic to the case; in many cases he will find that the cataract is removed by the treatment; but should he find that after the patient's general health is restored the cataract still resists the medicine, either wholly or partially, then, and not till then, will he recommend its surgical removal.

I propose, therefore, as an amendment, the following resolution:

"That in non-surgical cases we disapprove of medicated topical applications, and surgical operations or mechanical appliances in the place of the best indicated remedy, as being also non-homœopathic."

